# Why We Fail

**Published:** 1887-10

**Authors:** 


### HOMŒOPATHIC PHYSICIAN,

A MONTHLY JOURNAL OF MEDICAL SCIENCE.

"If our school ever gives up the strict inductive method of Hahnemann, we are lost, and deserve only to be mentioned as a caricature in the history of medicine."—CONSTANTINE HERING.

Vol. VII. OCTOBER, 1887. No. 10.

### WHY WE FAIL.

Some time ago this question was asked, and the answer given was that homœopaths of to-day fail to do such cure-work as the grand masters formerly did because we are lacking in knowledge of pathology and kindred branches. This answer does not account for the poor success of the modern homœopaths. Homœopathic physicians were never better educated in general medicine than they are to-day. The cause of all our failures lies in the one neglect of carefully individualizing our cases. The tendency is rather toward routine work and generalizing. We prescribe (and are taught to prescribe) too much for diseases rather than for patients. As a proof of this assertion and as a demonstration of the cause of our failures—failures due entirely to lack of homœopathic prescribing—we quote the following specimens of homœopathic *teaching*. These items are notes published in a journal to show what the colleges are doing. One can easily see how little Homœopathy is taught by such lecturers:

Professor P. recommends the use externally of the fluid extract of Sanguinaria in lupus. The hardened masses should first be removed with warm water, and then apply the Sang. Internally may be given Kali iod., but the Professor thinks the Sang. is sufficient.

Professor H. speaks very highly of Ipecac in many nervous diseases, especially of its use in cerebro-spinal meningitis; says it is not used often enough by the profession.

For diphtheria at puberty, where digestive disorders have been preceded with anæmia, etc., Professor W. recommends the use of

Ferrum acet. The digestive disorders will be found at the root of the trouble. The cutaneous symptoms, respiratory, motor, and sexual symptoms in order of importance.

For fibroid tumors of uterus Professor A. G. B. speaks very highly of the internal use of *Iodide of Lime*. Not the Calc. jod., but a preparation of *iodized lime*. He has cured numerous cases of uterine fibroids with this preparation:

℞ Iodide of Lime, grs. V.; Aquafortis, Qss. Dessertspoonful three times per day. Continue the use of the remedy for some length of time.

Professor F.'s favorite for the constipation of pregnancy is a tablespoonful of Flaxseed (whole seed), soaked over night in a half glass of cold water, this to be drunk by the patient on rising in the morning. Relieves over ninety-five per cent. of this class of cases. The amount of Flaxseed may be increased to two or three times the above measure.

Professor G. says that the use of the bandage and pinning-blanket as a part of the baby's dress is the prime cause of nine-tenths of all hernia, and furnishes a predisposing, if not an exciting, cause to gastritis and weakened heart action, weak lungs, and many other ills to which flesh, especially baby flesh, is heir. He advises the use of a light bandage, secured with two safety-pins, which is to be discontinued so soon as the cord has sloughed off. In twenty-two years he has a record of only one case of rupture resulting from this line of treatment, a case of hereditary syphilis which was "not made to hold together."

Professor F. also recommends in post-partum hemorrhage "a half ($\frac{1}{2}$) teaspoonful of Squibb's fluid extract of Ergot in water, and repeat the dose in fifteen minutes if necessary."

Professor of Practice recommends Gelsemium for impotency. No symptoms!

"While lecturing on the subject of Arsenicum album, Professor M. effectively advocated its use in typhoid fever *before* the supervention of extreme prostration, which is often waited for as an indication for this drug. He believes it is better to anticipate a little, and give it early." (Before indicated!)

Professor S. gave the following as a good corn-remover:

| | | |
|---|---|---|
| ℞.—Salicylic acid, . . . . . . . . . . . . . . . | grs. | xv. |
| Alcohol ext. Cannabis Indica, M., . . . . . . . | | viss. |
| Alcohol (95 per ct.), . . . . . . . . . . . | " | xv. |
| Ether, . . . . . . . . . . . . . . . . . . . | " | xxxviiss. |
| Flexible collodion, . . . . . . . . . . . . | " | xxxv. |

Apply night and morning.

Another case as reported is most amusing. A professor accidentally cures an ulcer by *internal* medication. The account reads:

"The Professor prescribed Thuja $^{6x}$ and ordered the tincture as local application. Three weeks later, when the patient returned with scarcely a vestige of the ulcer remaining, the Doctor admitted that he was inclined to be skeptical, and would fain have attributed the result, in part, at least, to local treatment, but when he learned that the patient had been provided with *no* local application, there being no tincture on hand, and that *no local treatment* had been resorted to, not even picking off the scab, he graciously acknowledged 'one more score for Thuja.'"

The Dean of one of these "truly homœopathic" colleges acknowledges he uses Morphine as a palliative and would be open to severe criticism if he did not continue to do so.

Why we fail: because we do not practice Homœopathy!

